# Study of the Ultraviolet Effect and Thermal Analysis on Polypropylene Nonwoven Geotextile

**DOI:** 10.3390/ma14051080

**Published:** 2021-02-26

**Authors:** Clever Aparecido Valentin, Marcelo Kobelnik, Yara Barbosa Franco, Fernando Luiz Lavoie, Jefferson Lins da Silva, Marta Pereira da Luz

**Affiliations:** 1 São Carlos School of Engineering, University of São Paulo-USP, São Paulo 13566-590, Brazil; cclever@sc.usp.br (C.A.V.); mkobelnik@gmail.com (M.K.); yarabf@usp.br (Y.B.F.); jefferson@sc.usp.br (J.L.d.S.); 2 Department of Civil Engineering, Mauá Institute of Technology, São Caetano do Sul 09580-900, Brazil; 3 Eletrobras, Furnas Centrais Elétricas S.A., Aparecida de Goiânia 74923-650, Brazil; martaluz@furnas.com.br; 4 Industrial and Systems Engineering Postgraduate Program (MEPROS), Pontifical Catholic University of Goiás (PUC Goiás), Goiânia 74605-010, Brazil

**Keywords:** thermal properties, polypropylene, nonwoven geotextile, thermal analysis, UV-aging

## Abstract

The use of polymeric materials such as geosynthetics in infrastructure works has been increasing over the last decades, as they bring down costs and provide long-term benefits. However, the aging of polymers raises the question of its long-term durability and for this reason researchers have been studying a sort of techniques to search for the required renewal time. This paper examined a commercial polypropylene (PP) nonwoven geotextile before and after 500 h and 1000 h exposure to ultraviolet (UV) light by performing laboratory accelerated ultraviolet-aging tests. The state of the polymeric material after UV exposure was studied through a wide set of tests, including mechanical and physical tests and thermoanalytical tests and scanning electron microscopy analysis. The calorimetric evaluations (DSC) showed distinct behaviors in sample melting points, attributed to the UV radiation effect on the aged samples. Furthermore, after exposure, the samples presented low thermal stability in the thermomechanical analysis (TMA), with a continuing decrease in their thicknesses. The tensile tests showed an increase in material stiffness after exposition. This study demonstrates that UV aging has effects on the properties of the polypropylene polymer.

## 1. Introduction

Geosynthetics are polymeric materials with growing use in civil engineering works. The geotextile is a geosynthetic usually manufactured with polypropylene or polyester, used for several functions, such as filtration, drainage, protection, separation, or reinforcement in a variety of applications (e.g., landfills, roadways, railways, ponds, retaining walls, or coastal protection structures).

Geotextiles have been used for the past thirty years for different types of containers, such as fabric forms for concrete paste, hand-filled sandbags, and other innovative systems involving containment of soils. Especially for dewatering high water content materials, these products are experiencing increasing market acceptance. These applications require geotextile exposure for months [1,2,3], and thus it is imperative the proper understanding of its long-term durability since geotextiles present high susceptibility to photo-initiated degradation due to its extensive surface area [4]. In some applications, the geosynthetic design life must be larger than 100 years, and thus its durability should be guaranteed. Therefore, a better understanding of the factors that influence the degradation processes and of the tools to estimate material durability is of most importance to preserve product performance at operation [5,6,7,8].

In general, geotextiles are exposed to ultraviolet (UV) light only during the installation time, which can be insufficient to cause relevant degradation. As an example, Faure et al. exhumed a polypropylene geotextile (over 20-years old) applied as a filter in Valcros dam and they did not find much degradation after its installation [9]. However, this does not guarantee a design lifetime of 100 years [7].

Polypropylene (PP) is the major polymer used to manufacture geotextiles over the world. Despite presenting poor resistance to oxidative degradation, PP resists well to some chemical substances. Photo-oxidation can promote the complex chain reaction mechanism involved in the oxidative degradation process of polypropylene. In fact, the long-term behavior of PP geotextiles is determined by its thermal oxidation resistance [6,7].

Oxidative degradation is an important mechanism of degradation related to the exposure to O_2_ from the environment, followed by the formation of OH and CO functional groups. To inhibit this process, which starts with a chain reaction propagated by chemical radicals, antioxidants or stabilizers are added to the polymer. These additives protect the resin during the service life of the product, and, without them, degradation would be so rapid that service life would be only of a few years for an unstabilized PP product [10,11].

Different methods are used to study the durability of geotextiles subjected to weathering, such as field and laboratory tests. The field method is restricted to the specific conditions of the site, and its duration can be quite long. Laboratory accelerated aging tests represent the boundary conditions from field applications and they can be required to understand the behavior of the material facing the oxidative exposition [7,9,12].

Previous studies have evaluated the thermal and oxidative behavior of polypropylene. Bertin et al. [13] examined the effects of thermo-oxidative degradation on the PP polymer. Gupta et al. [14], by using X-ray diffraction analysis and differential scanning calorimetry analysis (DSC), verified that the degree of crystallinity of polypropylene increased with longer UV exposure times, which the authors attributed to the progress of photo-oxidation and consequently the increase in the number of chain scissions. Carneiro et al. [15], in its turn, studied the synergism between degradation agents by conducting three degradation tests: immersion in liquids, thermo-oxidation, and artificial weathering. The authors analyzed the agents in isolation and in combination, and sometimes, the combined effect of two degradation agents was the most detrimental. Both thermo-oxidation and artificial weathering tests showed decreases in tensile resistance and elongation at maximum load, with higher reductions with artificial weathering.

Horrocks et al. [16] studied the influence of carbon black, a common UV-stabilizer, on the properties of polypropylene geotextile tapes. The authors detected increases in thermal stabilities when carbon black concentration increased from 2.5 to 5%. From DSC analysis, they noted that the crystallinity of the geotextile tapes slightly increased with oven aging for the unfilled and filled tapes. Further, carbon black increased considerably the UV durability of the filled tapes.

In the present study, the aim was to investigate the effects of accelerated aging in polypropylene (PP) nonwoven geotextile before and after 500 h and 1000 h exposure to ultraviolet (UV) light. A wide set of evaluations and tests were performed, including scanning electron microscopy, thermal analyses, kinetic evaluation from degradation, and mechanical tests. Previous studies have focused mainly on the effects of aging on the mechanical properties of the geotextile material, in a macro-scale approach, with little or no emphasis on the influence of UV-radiation in its thermal characteristics [4,5,12,15,16]. Therefore, this paper aims to contribute to deepening the learning on the effects of UV-radiation on the durability of geosynthetics, both in macro and micro-scale perspectives.

## 2. Materials and Methods

### 2.1. Geotextile and Accelerated Weathering Tests

This work studied a commercial polypropylene (PP) nonwoven needle-punched geotextile manufactured in Brazil with a nominal mass per unit area (MPA) of 400 g·m^−2^, with 1.0% (in mass) of light stabilizers and UV absorbers incorporated into the polymer during the geotextile manufacture. The product is composed of gray extruded cut fibers which are needle-punching in its manufacturing. Table 1 shows its main characteristics, determined from standardized tests [17,18,19,20].

It was used a UV-weathering chamber from Equilam, model EQUV with fluorescent UVA-351 lamps, manufactured by Philips, with a wavelength range from 295 nm to 365 nm, peak emission at 351 nm, and 40 watts’ power. The equipment was programmed to work in cycles of 8 h of UV light at 70 °C followed by 4 h of condensation at 50 °C for 500 h and 1000 h. The effects of the weathering were evaluated by comparing the virgin (reference) and aged samples by multiple techniques.

### 2.2. Mechanical Properties

Tensile tests [20] were performed for the virgin (Table 1) and aged samples in an EMIC Universal Machine, model DL 3000, manufactured at São José dos Pinhais, Brazil, with pneumatic grips and a 20-kN load cell. The test method covers strip test procedures to determine tensile strength and specimen elongation. Each test was carried out with five specimens (50 ± 0.5 mm wide) in the machine direction of production with a displacement rate of 300 ± 3 mm·min^−1^.

### 2.3. Scanning Electron Microscopy

Scanning electron microscopy (SEM) was performed with geotextile specimens of 5 × 5 mm randomly removed from the virgin and aged samples. It was used a ZEISS SIGMA microscope manufactured at Oberkochen, Germany, equipped with an Oxford X-ACT EDS/EDS detector and an electron acceleration voltage of 3.0 kV. Each specimen was fixed to the sample holder with a silver suspension followed by a gold deposit to ensure electrical conductivity.

### 2.4. Thermal Analysis Methodology

The thermoanalytical curves (thermogravimetric (TG), thermomechanical analysis (TMA), and differential scanning calorimetry (DSC)) were obtained to compare the thermal behavior of the samples subjected to different exposure times to UV radiation and to verify their thermal stability. The samples were cut into small pieces around 4 mm × 4 mm for TG and DSC analyses and 6 mm × 6 mm for TMA measurements.

For the TG-DTA (differential thermal analysis) and TMA curves, the following equipment was used, respectively: SDT 2960 and SDT 2940, both from TA Instruments, New Castle, DE, USA. DSC studies were conducted with a DSC1 Stare, from Mettler Toledo at Columbus, OH, USA.

TG-DTA analyses were performed atα-alumina crucible with heating rates of 10, 15, 20, and 30 °C min^−1^, under synthetic air and nitrogen gas purges, with the flow rate of 100 mL min^−1^. TMA analyses were conducted under nitrogen gas purges with a flow rate of 50 mL min^−1^ and a heating rate of 5 °C min^−1^. Evaluations of the melting point, crystallization, and glass transition in the DSC instrument were performed in an aluminum crucible under nitrogen gas purge with a flow rate of 50 mL min^−1^. In addition, at DSC, the evaluation was carried out under low temperature, with the samples being cooled from 200 to −80 °C and subsequently heated from −80 °C up to 200 °C, both at a cooling/heating rate of 10 °C min^−1^. However, to evaluate the glass transition, a heating rate of 30 °C min^−1^ was used. For the kinetic study, the Flynn–Wall–Ozawa (FWO) method was considered, with the use of four TG/DTG (derivative thermogravimetric) curves [21,22,23].

## 3. Results and Discussion

### 3.1. Thermoanalytical Analyses

#### 3.1.1. TG/DTG and DTA Analyses

**Figure 1:**
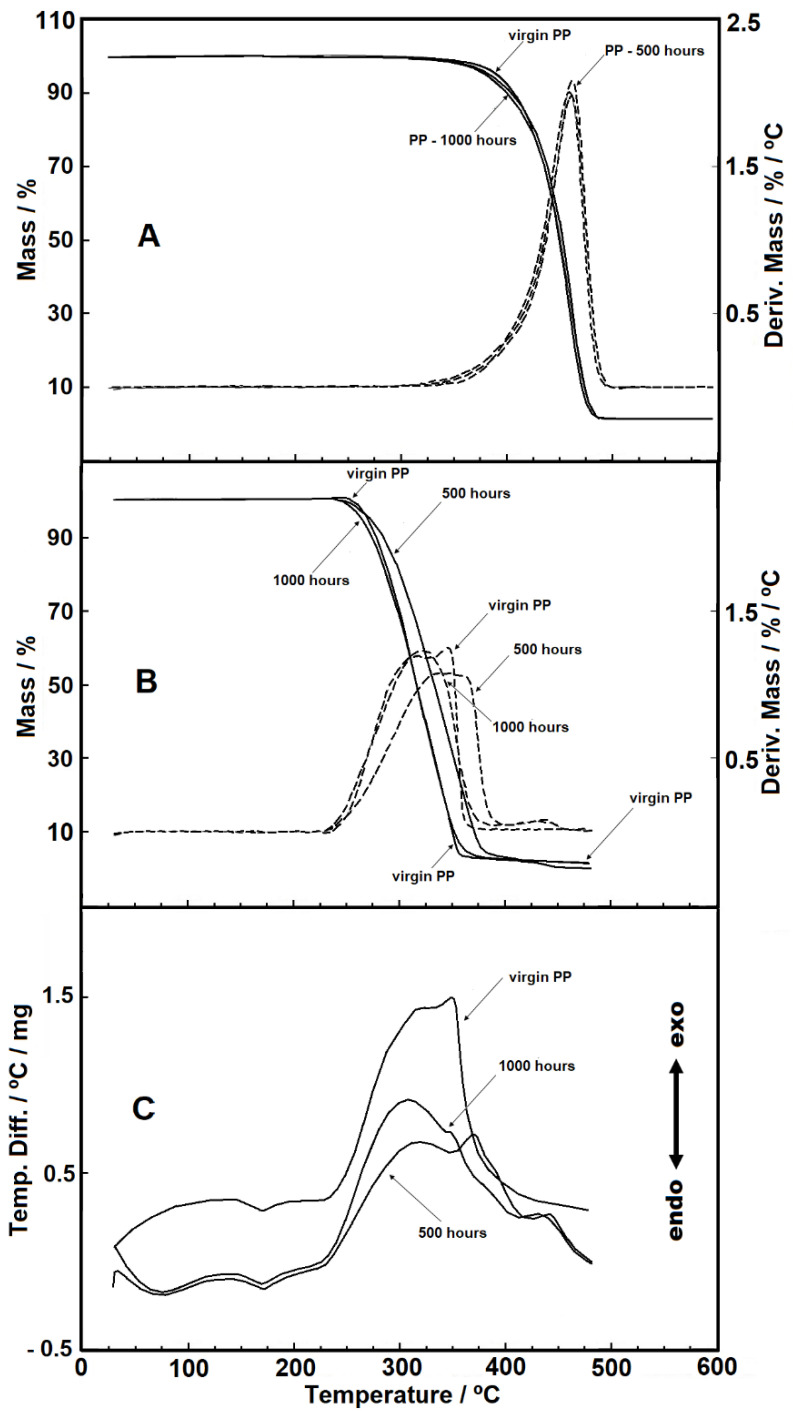
(**A**) Thermogravimetric (TG)/DTG curves in nitrogen gas purge, with sample masses around 3.50 mg; (**B**) TG/DTG curves in synthetic air gas purge, with sample masses around 3.20 mg and (**C**) DTA curves in synthetic air gas purge, with sample masses around 3.20 mg (flow of 110 mL min^−1^ in α-alumina crucible).

Figure 1A,B show the overlapped curves from TG/DTG analyses for the PP samples in synthetic air and nitrogen purge gases, respectively. In nitrogen (Figure 1A), the thermal behaviors occur in only one stage of thermal decomposition, with similar behaviors between samples. For the test in synthetic air (Figure 1B), a minor difference during the thermal decomposition took place. In this case, the samples aged for 500 h and 1000 h showed thermal decomposition beginning at 228 °C, which is less than the value for the virgin sample (235 °C). Furthermore, the DTG curves from synthetic air also show that the decomposition reaction does not occur in a single step for the virgin sample, being possible to identify an event during the last step of the main thermal decomposition.

At the end of the thermal decomposition for the analysis in nitrogen, we detected a dark carbonaceous residue impregnated in the crucible, removed by heating the crucible at an elevated temperature. For the analysis carried out in synthetic air, we spotted a residue similar to ash, easily removed when it was blown. Table 2 shows the temperature ranges related to the TG/DTG curves in synthetic air and nitrogen gas purges.

Figure 1C shows the DTA curves in synthetic air. Between 145 and 190 °C, an endothermic event takes place, which it is attributed to the melting of the samples, agreeing with the peaks seen in the DSC curves. Between 240 °C and 435 °C, an exothermic event attributed to the material oxidation can be observed. It is possible to see that a multi-step reaction occurs for all the samples since the DTA curve shows an enlarged base. Furthermore, for the samples subjected to 500 and 1000 h of UV exposure, an exothermic event between 430 and 460 °C takes place, related to the second thermal decomposition step of the samples.

#### 3.1.2. Kinetic Analysis

**Figure 2:**
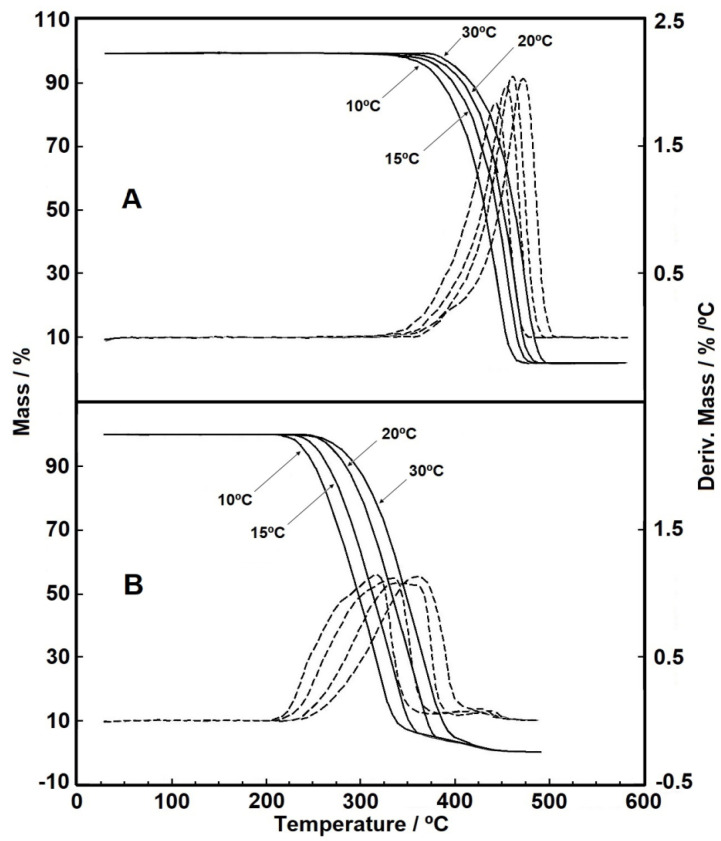
(**A**) TG/DTG curves for the virgin polypropylene (PP) in nitrogen gas purge, with sample masses around 3.50 mg; (**B**) TG/DTG curves for the 500 h PP sample in synthetic gas purge, with sample masses around of 3.20 mg. (all analyses conducted with heating rates of 10, 15, 20, and 30 °C min^−1^ with flow of 110 mL min^−1^ in α-alumina crucible).

Kinetic evaluation from the DTG curves data is shown in Figure 2A,B, in which four heating rates in nitrogen purge gas (virgin sample) and synthetic air (500 h sample) were used. The curves for the 1000 h sample presented similar behavior and are not reproduced here for brevity.

It is common knowledge that the comparison between DTG curves in inert gas and with increased oxygen pressure helps the interpretation of reaction kinetics since the main mechanism of oxidative degradation in polypropylene geotextiles is free radical, initiated by thermal cleavage of the polymer bonds [24]. Therefore, in Figure 2B the curves related to the 500 h sample were obtained in synthetic air (oxidizing atmosphere) for a better comparison with the results of the virgin sample in Figure 2A (nitrogen purge gas). It is possible to see that the increase in the heating rate caused a displacement between the curves, sustained throughout the thermal decomposition. The thermal displacement with rising in temperature is expected, but Valentin et al. [21] reported negligible displacements under different heating rates when studying the thermal behavior of HDPE (high-density polyethylene) geomembrane.

Ozawa–Flynn–Wall method (FWO) was used to estimate the activation energy by TG non-isothermal procedures. The thermal decomposition of a material can be mathematically described by the kinetic triplet (E, logA and f (α)). One of the relevant information of this method is the approach for the expression to Arrhenius integration equation. A solid-state reaction of Arrhenius can be expressed by the following general equation [25,26].

(1) $$\frac{d\alpha }{\mathrm{dT}}=\frac{A}{\beta }\exp\left(\frac{-E}{\mathrm{RT}}\right)f\left(\alpha \right)$$

where the fractional degradation (α) is temperature (T) dependent during an increase of heating rate (β). Since the FWO is an isoconversional method, the angular and linear coefficients of a plot of log versus 1/T at different and constant α give the E(α) and A values, respectively [27,28].

**Figure 3:**
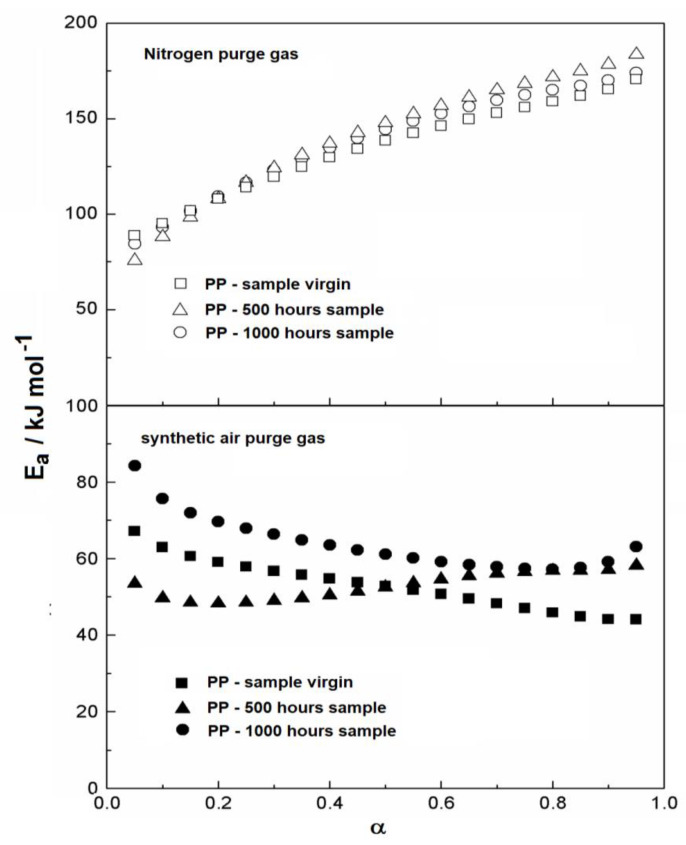
Activation energy (Ea) vs. conversion degree.

Table 3 shows the temperature intervals used for the kinetic data acquisition from the DTG curves and the mean values of activation energy with its respective correlation coefficient. As expected, the activation energies obtained in synthetic air were lower than those in nitrogen gas purge, because the reaction in the former favors material oxidation and therefore decreases the activation energy during decomposition. This behavior can be seen in a typical FWO plot, as shown in Figure 3, which portrays the E_a_ data vs. conversion degree (α) for the thermal decomposition intervals evaluated.

Observing the kinetic behavior of the samples evaluated in nitrogen (Figure 3), no distinct difference could be detected from the beginning up to the end of the thermal decomposition, which indicates a homogeneous reaction. However, for the analysis in synthetic air, it is possible to see that the initial values of activation energy differ from each other. The 1000 h sample showed the largest initial activation energy, whereas the lowest value occurred for the material exposed for 500 h. The virgin sample showed an intermediary value for the initial activation energy. Since the analysis in synthetic air promotes oxidation reactions itself, it is not possible to conclude that the effect of exposure to ultraviolet radiation had a significant effect on the activation energy values.

#### 3.1.3. DSC and TMA Analyses

**Figure 4:**
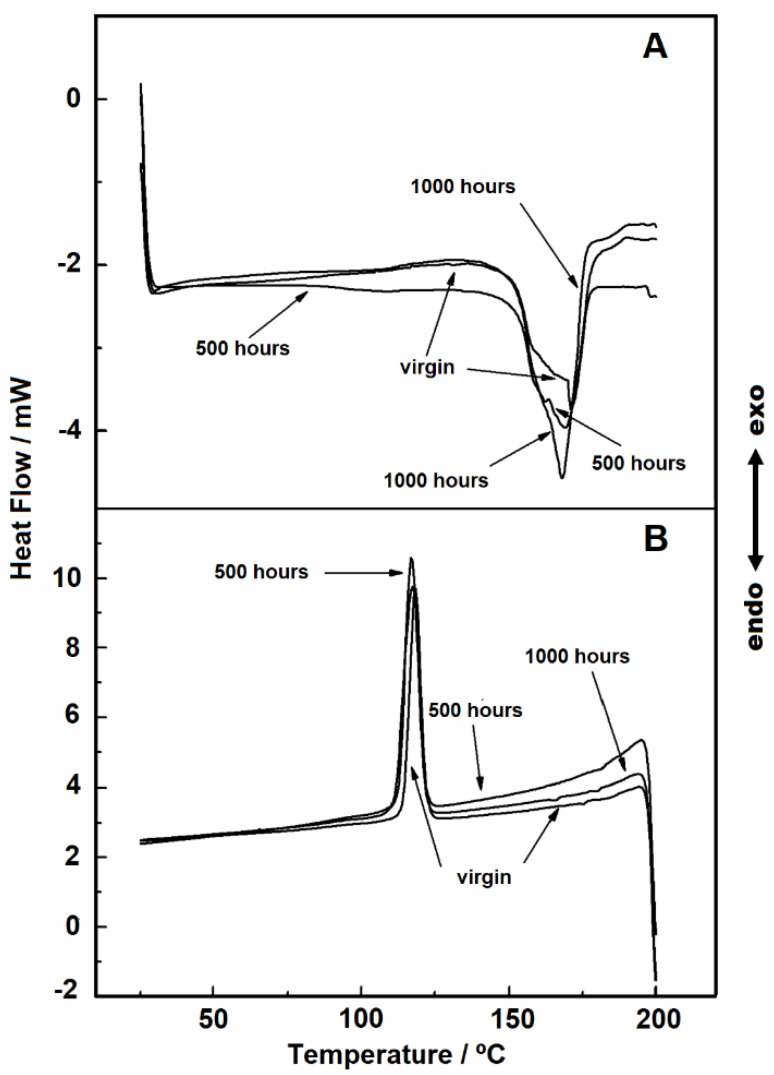
DSC curves with heating rate of 10 °C min^−1^ under nitrogen gas purge, flow of 110 mL min^−1^ in aluminum crucible, and sample masses around 3.50 mg: (**A**) heating and (**B**) cooling.

Figure 4 shows the DSC heating and cooling curves in the temperature range of 25 to 200 °C under nitrogen gas purge. During the heating, it is possible to observe an endothermic peak, due to the melting of the material. For the virgin sample, 500 and 1000 h samples, the melting peak has a wide peak area, which indicates overlapped reactions.

The values of enthalpy of fusion correlates to the endotherm peak area in the DSC curves, and they were 87, 78, and 80 J/g °C, for the virgin, 500 and 1000 h sample, respectively. Since enthalpy values are proportional to crystallinity no detectable change in crystallinity between the two aged samples (500 and 1000 h) was observed. This result is attributed to the material’s heating effect because, during the melting process, the PP fibers are fused again and therefore homogenized.

Gupta showed that with the increase in UV exposure time for pure PP and controlled PP/mLLDPE blend systems, there was an increase in the peak area and thus in crystallinity [14]. Similarly, Horrocks, et al. found that the DSC curves showed enlarged endotherm peak areas for oriented PP geotextile tapes containing carbon black of varied characteristics under UV exposure [16]. A decrease in crystallinity in both studies was observed, attributed to the breaking of polymeric chains and molecular reorganization. However, in the present study, we worked with a commercial nonwoven geotextile with composition and random fiber disposition that are likely affecting material response to UV exposure. Further research is therefore needed to better elucidate this behavior for nonwoven geotextiles.

Figure 4B shows the crystallization stage, in which it is possible to see that the peaks are similar. The melting point favors the disorder of the molecules, erasing the effects caused by exposure to UV radiation. In addition, as it is a nonwoven geotextile, the fusion causes the filaments to join. Thus, during recrystallization there is a molecular reorganization and therefore, the peaks will be similar.

**Figure 5:**
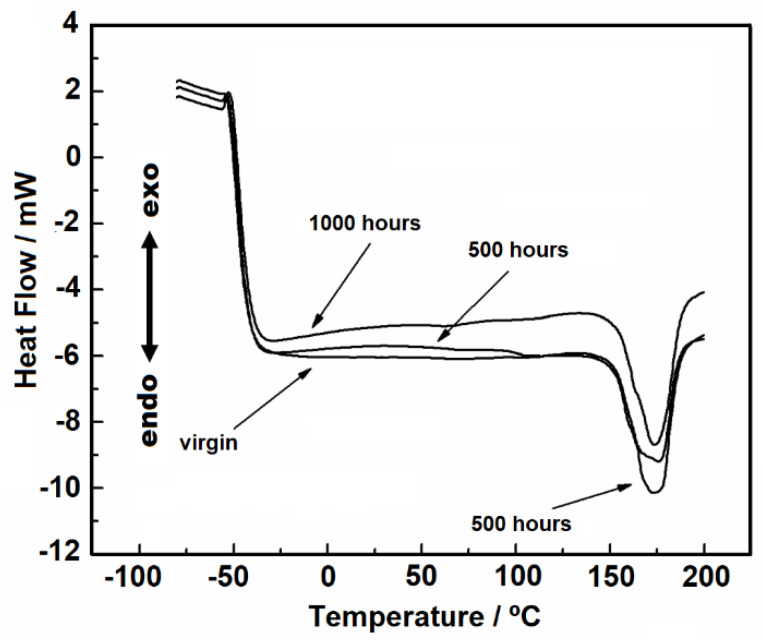
DSC curves used to evaluate the glass transition (heating rate of 30 °C min^−1^ under nitrogen gas purge with flow of 110 mL min^−1^ in an aluminum crucible and sample masses around 3.50 mg).

The evaluation of glass transition was conducted with a heat flow rate of 30 °C min^−1^, and its value would be expected to be in the temperature range of −20° to −5 °C [29]. However, we could not detect this for the studied samples (Figure 5), probably because of the material structure: a nonwoven geotextile with a randomly distributed pattern of fibers. Thus, given the distance of the wires and also the lack of sensitivity of the DSC cell, it was not possible to capture this event.

**Figure 6:**
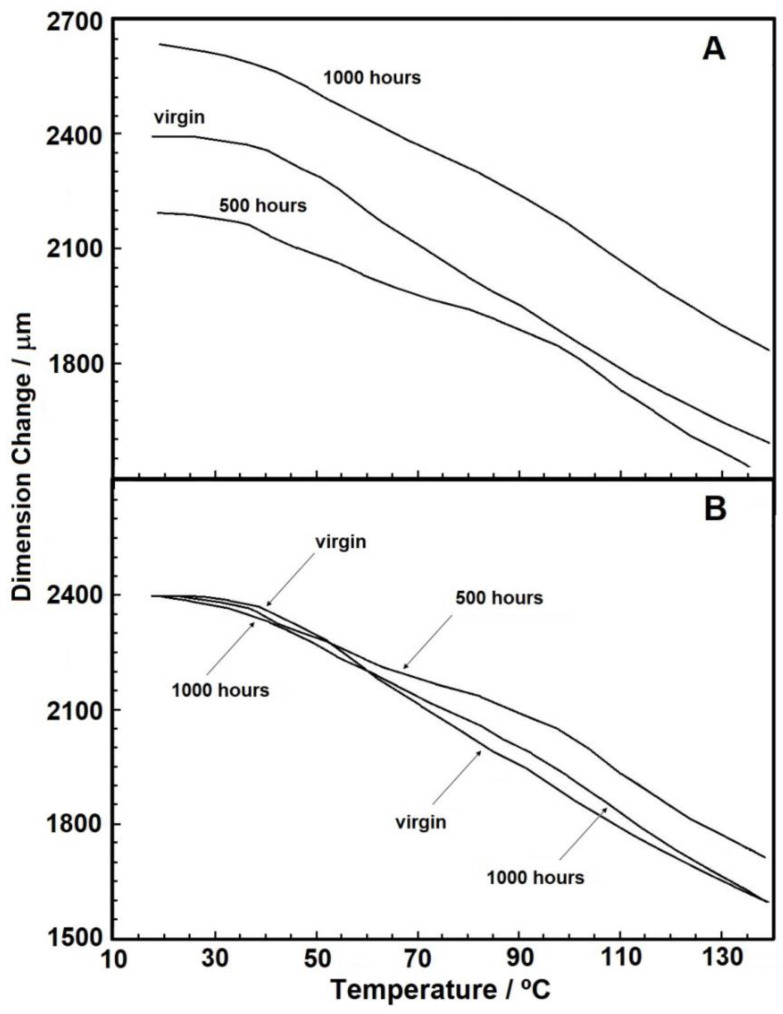
(**A**) Thermomechanical analysis (TMA) curves with a heating rate of 5 °C min^−1^ under nitrogen purge gas (**B**) overlapped normalized curves.

Figure 6A,B show the results for the TMA analyses (without applied force), in which the change in sample dimension is presented in micrometers (µm). A reduction in sample dimension can be observed for all samples, which we attributed to the interlacing of the material fibers that get loose under heating and tend to get closer to each other. It is worth mentioning that each sample has a different nominal thickness since the manufacturing process for nonwoven geotextiles cannot keep a precise thickness for the product. For this reason, as seen in Figure 6A, the larger thickness for the 1000 hours sample is not related to the UV radiation effect, but rather to product variability.

Figure 6B shows the normalized thickness values for comparison, in which it is possible to see that after 60 °C a slight difference between the samples’ behavior occurs. In absolute values, the virgin sample experienced a thickness reduction of 27.91%, while values of 24.65% and 26.71% were obtained for the 500 and 1000 h samples, respectively. These results show that the difference in total dimensional between the samples was small. Regarding the application of PP geotextile material in engineering works, despite the minor differences among the samples, the reductions relative to the initial dimensions were significantly high. Therefore, it can be concluded that this material is susceptible to heating effects when stored in sheds under elevated temperatures, presenting relevant dimensional changes.

### 3.2. Scanning Electronc Microscopy

**Figure 7:**
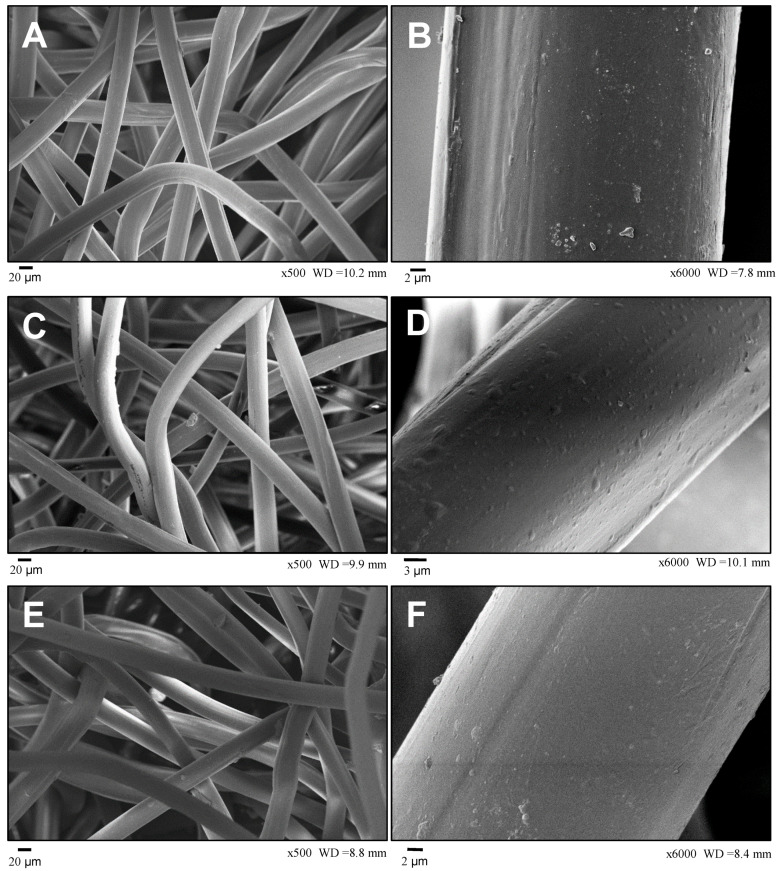
Scanning electron microscopy photographs of the PP fibers: (**A**,**B**) virgin PP; (**C**,**D**) PP 500 h; (**E**,**F**) PP 1000 h. (**A**,**C**,**E**) with 500× magnification and (**B**,**D**,**F**) with 6000× magnification.

SEM analysis did not show significant changes in PP fiber surface after exposure to 500 h and 1000 h of artificial weathering, as seen in Figure 7 for magnifications of 500× and 6000×. Therefore, the tested exposure times did not cause apparent fiber damage, a sign that the amount of stabilizer added in the manufacturing process of the PP geotextile (information not usually disclosed by manufacturers) was effective in preventing the fiber degradation, at least from the perspective of visual surface fiber damage and for the selected exposure times.

It is worth noting, however, that UV-stabilizers does not prevent permanently the degradation, it only delays it. The photo-stabilizers protect the polymer against degradation by absorbing the energy from excited free radicals that would otherwise react with the polymer chains. This protection will last until the stabilizer is fully consumed, after which the polymer is exposed [4]. Therefore, care should be taken when using PP geotextiles in applications in which the exposure time can be significant since non-stabilized PP material present poor weathering stability [24]. In fact, Carneiro and Lopes [30] have found extensive damage in unstabilized PP fibers due to a year of outdoor exposure, findings captured by SEM photographs that clearly showed the breakage and transverse cracking of the fibers.

### 3.3. Tensile Properties

**Figure 8:**
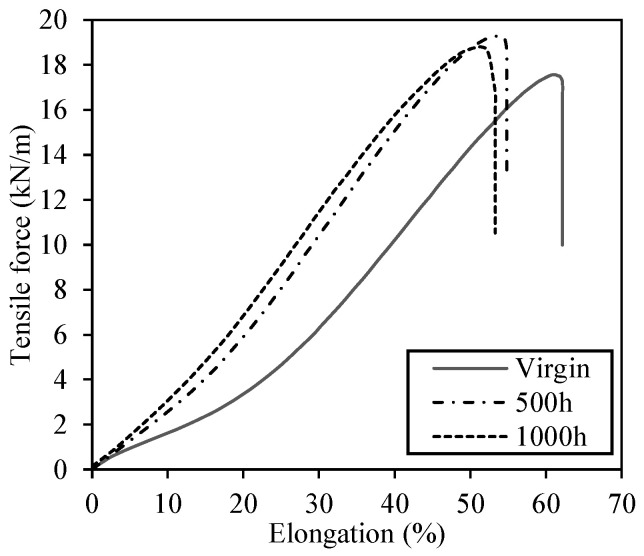
Mean “tensile force–elongation” curves obtained for the PP geotextile before and after accelerated weathering for 500 h and 1000 h (machine direction).

Table 4 presents the test results of the tensile tests conducted for the aged samples. Figure 8 shows the mean tensile force–elongation curves for each exposure time and the reference sample (virgin). We can see an increase in tensile resistance and a decrease in tensile elongation for the aged samples compared to the virgin one. The 500 h sample and 1000 h samples had increases of 10% and 8%, respectively, for the tensile resistance and decreases of 13% and 16%, respectively, for the tensile elongation at break. Therefore, polymer morphologic changes caused by UV exposition can explain the material’s stiffness increase after exposure. Similarly, Gupta et al. [14] have found a decline in elongation at break for PP/mLLDPE blend samples exposed to UV radiation for a period of six weeks (1008 h), which the authors attributed to the extensive chain scission events, detrimental to polymer ductility, due to degradation.

The stiffness increases in reference elongations of 2% and 5% were in the order of 11% and 33% for the 500 h sample and of 40% and 50% for the 1000 h sample, respectively. This increase in strength was attributed to the increased stiffness of the material during exposure. For pure polypropylene samples and oriented PP tapes with carbon black, the increase in stiffness has been correlated with an increase in material crystallinity after UV exposure ([14,16]). However, in the present study, DSC analysis results did not present significant changes in crystallinity as a function of UV exposure time. Considering that the amount of sample used in DSC analysis is minimal, this apparent inconsistency may be explained by the fact that we are dealing with a commercial nonwoven geotextile which composition and random fiber disposition may influence material response to UV exposure, which warrants further study.

Nonwoven geotextile fibers are randomly oriented in the product. In the case of a needle punching geotextile, a set of hundreds of specially designed needles punch a web of filaments, reorienting them to achieve mechanical bonding [31]. Therefore, the final product is an entanglement of filaments and not a continuum material such as film type materials, case of geomembranes. Hence, it is possible that UV radiation influences the interaction between filaments, which in its turn could explain the increase in material stiffness after exposure. Nonetheless, the verification of this hypothesis is necessary and it will be the focus of future studies.

A similar increase in PP geotextile stiffness was found in outdoor weathering tests conducted by Carneiro and Lopes [30], which the authors attributed to the accumulation of dust and dirt between the material fibers. In their study, though, the tensile resistance and elongation at rupture for exposure times from 6 to 36 months were smaller than those for the unexposed sample. Contrastingly, Carneiro et al. [15] results showed an opposite behavior, with a decrease in stiffness after artificial weathering. In their study, however, they used a smaller exposure time of 250 h.

## 4. Conclusions

This study used different analyses to identify the possible durability problems related to geotextile exposure to ultraviolet light, both with macro and micro-scale approaches. The UV-aging tests for 500 h and 1000 h of exposure led to degradation of the commercial PP geotextile tested.

Thermal analyses were conducted to identify material conditions before and after UV-exposure. TG curves showed slight changes in material thermal stability after weathering, which was also verified from the kinetic data in the two purge gas conditions studied. Temperature increase had a relevant influence on material thickness in the TMA analyses, though the differences between the UV-exposure times were negligible. From the DSC analyses, it was not possible to identify the effect of UV radiation on the samples melting or crystallization point, with similar endotherm peak areas indicating similar crystallinity independent of exposure time. Despite showing similar enthalpies, the samples showed apparently distinct behaviors for the melting peaks.

The tensile tests showed an increase in material stiffness after UV radiation exposure, with larger tensile strength and smaller elongation at the ultimate load. This behavior was attributed to a likely effect of UV radiation on the arrangement and interaction between filaments, which could explain the increase in material stiffness after exposure. This hypothesis, though, requires further studies to be better elucidated.

The evaluation of SEM photographs could not detect any significant superficial damage to the geotextile fibers after the artificial weathering. This is an indication that the durations of exposure evaluated were not sufficient to cause apparent damage in the fiber surfaces, and the amount of UV-stabilizers added to the material composition could protect the geotextile from superficial damage. However, the tensile resistance analyses indicate that there was an increase in material stiffness after UV exposition. Therefore, caution should be taken when exposure times are expected to be significant during construction or the long-term life of the application, especially in countries of tropical climates, with high radiation intensities and temperatures. Designers should then recommend appropriate measurements to reduce exposure times.

## Figures and Tables

**Table 1:** Main characteristics of the polypropylene nonwoven geotextile.

MPA (g m^−2^)	Thickness (mm)	Puncture Resistance (N)	Tensile Resist./(kN m^−1^)	Tensile Elong./(%)
412.3	3.58	729.54	17.91	62.92
(±5.95)	(±0.07)	(±107.21)	(±0.99)	(±2.35)

In brackets are the standard deviations.

**Table 2:** Temperature intervals (°C) obtained from DTG curves for the thermal decomposition steps in nitrogen and synthetic air gas purges, with a heat flow rate of 20 °C.min^−1^.

Compound/Purge Gas	Stage	∆T (°C)	Mass Loss (%)
Virgin PP (synthetic air)	1	235–369	98.6
	residue	---	1.4
500 h (synthetic air)	1	228–396	97.25
	2	396–478	2.75
	residue	---	0
1000 h (synthetic air)	1	228–384	97.45
	2	384–478	2.55
	residue	---	0
Virgin PP (nitrogen)	1	350–397	97.24
	residue	---	2.76
500 h (nitrogen)	1	331–498	97.52
	residue	---	2.48
1000 h (nitrogen)	1	315–498	99.14
	residue	---	0.86

**Table 3:** Temperature intervals used for the kinetic analysis and mean activation energy values (Ea).

Purge Gas and Sample	Temperature Ranges for Kinetic Evaluation (DTG Curves)	*E_a_/kJ mol^−1^	*r
Synthetic air Virgin PP	(10 °C) 224–316 °C	53.20 (±0.13)	0.97959
	(15 °C) 231–338 °C		
	(20 °C) 237–366 °C		
	(30 °C) 252–375 °C		
Synthetic air PP 500 h	(10 °C) 208–361 °C	53.10 (±0.08)	0.97659
	(15 °C) 214–378 °C		
	(20 °C) 230–398 °C		
	(30 °C) 238–409 °C		
Synthetic air PP 1000 h	(10 °C) 222–348 °C	64.08 (±0.11)	0.99965
	(15 °C) 230–367 °C		
	(20 °C) 231–379 °C		
	(30 °C) 239–396 °C		
Nitrogen Virgin PP	(10 °C) 342–477 °C	134.00 (±0.18)	0.99591
	(15 °C) 360–491 °C		
	(20 °C) 380–496 °C		
	(30 °C) 392–505 °C		
Nitrogen PP 500 h	(10 °C) 334–482 °C	141.11 (±0.22)	0.97306
	(15 °C) 341–490 °C		
	(20 °C) 365–495 °C		
	(30 °C) 392–505 °C		
Nitrogen PP 1000 h	(10 °C) 323–478 °C	140.70 (±0.19)	0.99976
	(15 °C) 335–492 °C		
	(20 °C) 349–495 °C		
	(30 °C) 372–509 °C		

**Table 4:** Tensile test results of the aged samples.

Aged Sample	Tensile Resist./(kN m^−1^)	Tensile Elong. at Break/(%)
PP 500 h	19.63	54.77
	(±2.77)	(±2.14)
PP 1000 h	19.27	52.69
	(±1.98)	(±2.93)

In brackets are the standard deviations.

## Data Availability

The data presented in this study are available on request from the corresponding author.
